# The processes of hospital discharge and recovery after blunt thoracic injuries: The patient’s perspective

**DOI:** 10.1002/nop2.929

**Published:** 2021-05-18

**Authors:** Edward Baker, Andreas Xyrichis, Christine Norton, Philip Hopkins, Geraldine Lee

**Affiliations:** ^1^ Florence Nightingale Faculty of Nursing, Midwifery and Palliative Care King’s College London London UK; ^2^ Emergency Department King’s College Hospital NHS Foundation Trust London UK; ^3^ Department of Intensive Care Medicine King’s College Hospital NHS Foundation Trust London UK

**Keywords:** analgesia, chest trauma, hospital discharge, hospitalisation, injury, medicines safety, nursing, patient pathway, rib fractures, trauma

## Abstract

**Aims:**

The aim of this study was to explore hospital discharge processes and the self‐management of recovery in the early post‐discharge period after blunt thoracic injury from a patient perspective.

**Design:**

Qualitative interview study.

**Methods:**

Interviews were conducted with participants recruited from 8 sites across England and Wales between November 2019–May 2020. Semi‐structured interviews were conducted between 5–8 weeks after hospital discharge, and in total, 14 interviews were undertaken. These interviews were recorded, transcribed and analysed using thematic coding.

**Results:**

Three main themes were identified from the analysis: (a) challenges in the discharge process, (b) coping at home after discharge and (c) managing medications at home. Pain was a dominant thread running throughout all themes which represented an important quality and safety concern for all participants. Associated concerns included insufficient preparation and education for hospital discharge, ineffective communication and subsequent unsafe use of opioids at home highlighting unmet patient care needs.

## 1 INTRODUCTION

For patients with blunt thoracic injury (BTI) presenting to major trauma services globally, recovery following discharge from hospital remains a challenging process (Marasco et al., 2015). BTI is defined as injury to the bony or soft tissues of the thorax or underlying organ systems caused through a blunt mechanism of injury (Baker & Lee, 2016). For younger individuals with BTI, common mechanisms include high velocity impact mechanisms (e.g. Falls from heights, road traffic collision etc.), whilst for older individuals, substantial injuries can also be sustained from simple low velocity mechanisms (e.g. falls from standing) (Kourouche et al., 2018). Significant physical, psychological and socio‐economic sequelae have been reported (Baker et al., 2018). It is likely that insufficient supportive care in the early post‐discharge period contributes to the burden experiences by this patient population. Hospital discharge describes a transition where hospital care ends and responsibility for ongoing care needs is transferred onto other healthcare providers (primary care, social care services and domestic environments) and is an opportunity where patients can be prepared to optimize their own recovery at home (Johnson et al., 2012; Markiewicz et al., 2020; Waring et al., 2014, 2019). There is a gap in the current trauma evidence base around the impact of the discharge process after BTI on early self‐management and recovery at home. It is not possible for clinicians to optimize recovery for this patient group where there is little understanding of the patient experience in this recovery phase.

## 2 BACKGROUND

Previous qualitative research in non‐trauma populations identified several factors that often negatively impact on the discharge process (Waring et al., 2019). These included poor communication between health and social care, lack of assessment and planning, inadequate notice of discharge, inadequate involvement of the patient and family, over‐reliance on informal care and lack of attention to the special needs of vulnerable groups (Waring et al., 2014). Although there has been substantial organizational work to improve the discharge process in high‐risk patient groups over the past decade, there is a paucity of research into the patient's experience and the effectiveness of the discharge process for trauma patients with BTI in the UK (El‐Eid et al., 2015).

In the general trauma population, previous research exploring the patient's transition from hospital care to manage their recuperation at home highlighted that inadequate knowledge and experience negatively impacts on an individual's ability to cope at home (Goldsmith et al., 2018a, 2018b; Kellezi et al., 2020; Sleney et al., 2014). Pain has been highlighted as contributing to patients not managing well at home following discharge with insufficient guidance, information and education on pain management impacting their self‐management (Goldsmith et al., 2018a; Gualandi et al., 2019). Despite these findings, there is currently insufficient knowledge around the impact of hospital discharge on recovery and self‐management after BTI and without this knowledge, it is not possible to critically review pathways for this patient group. The aim of this exploratory qualitative study was to describe the discharge and early post‐discharge recovery experiences of patients with BTI.

## 3 METHODS

### 3.1 Design

A qualitative study using semi‐structured telephone interviews was conducted. This manuscript has been developed following the Standards for Reporting Qualitative Research (SRQR) (O'Brien et al., 2014).

### 3.2 Study setting

The study included eight geographically diverse sites across England and South Wales including both urban, suburban and rural areas. All sites were UK National Health Service hospitals that were receiving hospitals for major trauma patients. This study is a qualitative component of a mixed‐methods study. Table 1 presents the context of the individual recruiting sites.

**TABLE 1:** Recruiting site characteristics

	Geographical location	Geographical population type	Population size	Trauma network status	Number of inpatient beds
Site 1	South Wales	Sub‐urban/rural	c. 390,000	Trauma Unit	750
Site 2	Greater London	Urban	c. 2.5 million	Major Trauma Centre	845
Site 3	Northern England	Sub‐urban/rural	c. 600,000	Trauma Unit	1,159
Site 4	Northern England	Sub‐urban/rural	c. 236,000	Local Emergency Hospital	500
Site 5	Greater London	Urban	c. 2.6 million	Major Trauma Centre	1,300
Site 6	South‐west England	Urban	c. 900,000	Major Trauma Centre	996
Site 7	South Wales	Sub‐urban/rural	c. 600,000	Trauma Unit	774
Site 8	South Wales	Rural	c. 600,000	Local Emergency Hospital	420

### 3.3 Study sample and recruitment

Between November 2019–May 2020, semi‐structured qualitative interviews were conducted with fourteen participants who had been admitted to hospital with BTI. Interviews were undertaken 5–8 weeks following hospital discharge. All participants were recruited into the “Rib Injury Outcomes Study” (RIOS) which aimed to investigate changes in Health‐Related Quality of Life and pain‐related outcomes in patients with blunt thoracic injuries over six months after hospital discharge. To provide context to this current publication, the quantitative components of this approach identified substantial levels physical burden of BTI during the first six months after hospital discharge which related to the development of chronic and neuropathic pain states. This had an overall negative impact on individual HRQoL over the first six months after hospital discharge with BTI. Eligibility for inclusion into RIOS was as follows:

Inclusion Criteria:

- Aged 16 years or above
- Admitted to a trauma receiving hospital with BTI
- Admitted to hospital for a period of 24 hr or greater

Exclusion Criteria:

- Acute/unstable spinal fracture or spinal cord injury
- Traumatic brain injury with cognitive impairment
- Altered mental status

Patients were recruited by clinical research staff at each site during the initial inpatient admission where informed consent was taken. Patients were purposively selected for interview from those patients who agreed to be interviewed during initial recruitment. Patients who initially agreed were contacted by EB after discharge from hospital to discuss participation. A sampling framework was developed focussing on factors including patient age, gender, geographical location and indicators of injury severity. The study aimed to use a maximum variation sampling approach using the factors introduced above. Participants were offered choices in relation to how interviews would be conducted (at home, hospital site or via telephone). Ninety‐two participants initially showed interest, and 14 eventually participated. No participants contacted withdrew from the study.

### 3.4 Data collection

All interviews were undertaken by the one interviewer (EB) who is an experienced Registered Nurse in Emergency Care currently undertaking a clinical doctoral research fellowship but was not directly involved with the provision of care for any participants. Interviews were audio recorded with permission and transcribed verbatim by a professional transcription service. Transcribed interviews were anonymised and reviewed with audio to check for accuracy and consistency.

The semi‐structured interview topic guide was developed using the literature on qualitative interviewing techniques and previous qualitative work on recovery after BTI (Claydon et al., 2017; Sleney et al., 2014). These focussed interviews were flexible in length but aimed to be between 15–20 min in length to minimize the burden on the participant. Table 2 presents the four broad topics covered in the interviews. Questioning was largely open‐ended and where appropriate; participants were given flexibility to lead and direct the discussion.

**TABLE 2:** Interview topic guide

Topic no.	Topic	Example questions/prompts
	Introductions	Are you happy to continue with the interview today? Do you have any questions?
1	The participant's injury	Can you tell me about your injury and how it happened?
2	Discharge Planning	During your admission to hospital, what information did you receive about planning your discharge? How did the healthcare team involve you in planning you discharge from hospital? What written information and advice were you given prior to your discharge from hospital?
3	Managing symptoms at home	Did you experience any symptoms from the BTI in the first month after discharge from hospital? How prepared were you for managing your own recovery at home after discharge from hospital? What were the main challenges you had to overcome during the early post‐discharge period?
4	Reflecting on your discharge	In what ways did you feel prepared for discharge from hospital? Were there any aspects of your discharge that you feel could have been done differently to help you cope at home?
	Conclusions	Do you have anything further you would like to add? Is there anything that you would like to ask me?

### 3.5 Data analysis

Data analysis was undertaken using a process of reflexive thematic analysis using an inductive approach (Braun & Clarke, 2006, 2019). Interview transcripts were uploaded into NVivo v.11 (QSR International Ltd), and initial data analysis was undertaken by EB. Subsequent discussion of codes and themes with GL, AX and CN resulted in a consensus on the names and definitions of codes, sub‐themes and themes. Table 3 presents the six‐stage approach to thematic analysis.

**TABLE 3:** Six‐stage approach to Thematic Analysis (V Braun & Clarke, 2006; V. Braun & Clarke, 2019)

	Title	Brief description of stage
Stage 1	Familiarizing yourself with your data	Transcription Reading and Re‐reading Noting initial concepts and ideas
Stage 2	Generating Initial Codes	Coding interesting features of the data in a systematic manner and collating data relevant to each code
Stage 3	Searching for Themes	Collating codes into potential themes, gathering all data relevant to each potential theme
Stage 4	Reviewing Themes	Checking that the themes work in relation to the coded extracts and the entire data set
Stage 5	Defining and naming Themes	Ongoing analysis to refine the specifics of each theme, and the overall story that the analysis tells, generating clear definitions and names for each of the themes
Stage 6	Producing the Report	The final opportunity for analysis. Section of vivid, compelling extracts, relating back to the analysis of the research question and literature, producing a scholarly report of the analysis

Thematic analysis focussed on identifying, examining and recording patterns in the data but also allowed for the abstraction and theorizing of themes from the data set. These patterns were important for describing the participants’ experiences of recovery becoming “units of meaning” in the analysis. Themes were identified by drawing together components or fragments of participants’ ideas or experiences which although meaningless when viewed in isolation, when combined, they form a comprehensive picture of the collective experiences of the participants (Braun & Clarke, 2006).

### 3.6 Trustworthiness and rigour

Rigour was maintained throughout by achieving trustworthiness criteria which have been used to demonstrates that data collection was conducted using precise, consistent and an exhaustive approach (Nowell et al., 2017). During data collection, participants were encouraged to lead the conversation and discuss topics candidly. To ensure accuracy, dependability and credibility, prior to terminating the interview, EB summarized key discussion points from the interview to ensure participant's statements were accurately understood in the context and enable participants to elaborate further on key areas of personal interest.

To ensure transparency and rigour through the data analysis process, the data were transcribed professionally and checked for accuracy by a member of the research team (EB). Initial analysis was undertaken by one member of the research team (EB) as this study forms a component of a PhD research study and this was followed by an in‐depth discussion of the data with GL formulating codes, sub‐themes and overarching themes. Further discussion around definitions of themes and codes between EB, GL, AX and CN and subsequently code and theme names and definitions were confirmed. Both field notes and a self‐critical reflexive journal were maintained throughout the data collection and analysis process to provide a clear audit trail of thoughts, decisions and choices made in these stages of the study.

## 4 FINDINGS

Of the fourteen participants with BTI recruited 10 were male, the predominant mechanism of injury was a fall of less than two metres. Study interviews length varied from 12–42 min. Although all participants were polytrauma patients and therefore had injuries in more than one body system, in all cases the BTI was the primary injury of concern. In all cases, extra‐thoracic injuries were classified as minor or moderate (1 or 2) using the Abbreviated Injury Score (AIS) (Baker et al., 1974). Table 4 presents participants’ demographic profile developed during the data collection process. Further example quotations are presented in File S1.

**TABLE 4:** Participants’ demographic profile

Participant Pseudonym	Age (years)	Gender	Geographical location	No. of Rib Fractures	Mechanism of Injury	Hospital Stay (days)	Extra‐thoracic injuries
Bill	65	M	South‐West England	6	Fall < 2 m	9	Yes
Robert	61	M	North England	5	Fall > 2 m	7	Yes
Stephen	70	M	South Wales	7	Fall < 2 m	10	Yes
Sally	52	F	South‐West England	6	Kicked by horse	5	Yes
Calvin	50	M	South Wales	7	Fall > 2 m	5	Yes
Lydia	48	F	Greater London	2	Pedestrian hit by vehicle	3	Yes
Oliver	62	M	North England	4	Fall < 2 m	5	Yes
Temi	71	F	Greater London	16	Fall > 2 m	6	Yes
John	62	M	North England	8	Fall > 2 m	8	Yes
Reg	77	M	South‐West England	6	Fall < 2 m	10	Yes
Gary	86	M	Greater London	5	Fall < 2 m	12	Yes
Karen	70	F	South Wales	2	Fall < 2 m	9	Yes
Richard	78	M	South Wales	2	Fall < 2 m	6	Yes
Henry	60	M	North England	1	Fall < 2 m	2	Yes

Three main themes were identified from the analysis: (a) challenges in the discharge process, (b) coping at home after discharge and (c) managing medication at home. Table 5 presents the themes, sub‐themes and codes identified during data analysis.

**TABLE 5:** Themes and codes table

Themes	Sub‐themes	Codes
Challenges in the discharge process	Suboptimal care co‐ordination in the interprofessional team	Issues in care coordination
		Issues in communication
		Understanding of discharge reasoning
		Lack of patient and family involvement
	The mismatch between the patient's expectations and their actual experience of the discharge process	Sharing of discharge information
		Insufficient written advice and guidance
		Being unprepared for early self‐management
		Leaving hospital
Coping at home after discharge from hospital	Optimizing recovery	Depending on others
		Daily life and activities
		Optimizing sleep and positioning at home
		Impact of extra‐thoracic injuries
		Accessing follow‐up care
	Living with symptoms after discharge	Mobility issues
		Shortness of breath
		Fatigue
		Pain and Neuropathic pain
		Aggravated pain
Using pain relief at home	Medication supply	Taking medications home
		Accessing further medications
	Medication safety	Opioid overdose
		Weaning from analgesics
	Side effects and concordance	Opioid constipation
		Neuro side effects of opioids

### 4.1 Theme 1: Challenges in the discharge process

Two main sub‐themes were identified which highlighted challenges patients experienced in the discharge process: (a) suboptimal care co‐ordination in the interprofessional team; and (b) the patients’ expectations of the discharge process.

#### 4.1.1 Subtheme 1.1: Suboptimal care co‐ordination in the interprofessional team

Participants stated that the number of professional groups involved in discharge planning was sometimes overwhelming and participants often perceived these professional groups as working in silos rather than a team approach. Participants identified how these professional groups’ input was either a facilitator or barrier to their discharge from hospital and identified components of their discharge that could have been improved or done differently:

…I think probably I would have also liked some psychological support when I came out… it triggered other traumas from my past that I hadn’t expected… [Lydia 48]

The complexities of the siloed approach to trauma care in the discharge process resulted in confusing and conflicting advice from different team members. One participant reported:

…I was given conflicting advice twice a day, from day 5 probably… [Bill 65]

Participants perceived that the discharge process and decision‐making was based predominantly on mobility assessment or on operational factors and bed pressures:

…obviously some trauma patient needed my bed. [Karen 70]

Many participants felt like they had a role in their own discharge process but for some, their voice was not heard or integrated into the process:

…I said well I don’t really think I’m able to go home because I can’t get out of bed. They weren’t interested…they just wanted to get me out and about and that was it. [Reg 77]

For others, the “desire to get home” clouded the opportunities to prepare themselves for self‐management at home:

I think my desire to leave overrode my fear and yes… I did feel fragile… not walking very well anyway… combine that with every movement being incredibly painful… [I was] absolutely exhausted at that point but I was just like yes, I want to go home… [Bill 65]

Participants appear to interpret their discharge and the actions of the interprofessional team as a passive process purely associated with leaving hospital but often do not appreciate the importance of discharge planning in optimizing their recovery at home.

#### 4.1.2 Subtheme 1.2: The mismatch between the patient's expectations and their actual experience of the discharge process

Almost all participants had clearly identified expectations of the discharge process. Several highlighted that discharge was not discussed until they were medically fit to go home resulting in a rushed process that focussed on them leaving hospital rather than preparing them:

It was at breakfast and they just said how are you feeling today and I said fine, I think I’m OK to go today …I organised someone to come and pick me up… The nurses came round and then she was like oh right I see you are going; I’ll just go and get your drugs for you and what you need. [I] signed a couple of forms and I didn’t see anybody else again. [Sally 52]

Several participants highlighted that the reality of discharge was not what they had expected resulting in them being both unprepared and apprehensive about leaving hospital where their care needs were consistently met:

I think when you leave hospital you are still in shock… I didn’t realise what the pain levels [would be like] … I think I was in denial about a lot of things. [Lydia 48]

You do have a bit of apprehension actually at that point because when you are in hospital you are in a safe place if something goes wrong. [Calvin 50]

The experience of leaving the hospital at the end of the discharge process also varied.

I was sent from the ward down to a discharge lounge and it took an hour and a half to get off the ward … It was made worse by the fact that the discharge lounge people had told me that my medication was ready after 3 hours but nobody from the pharmacy could be bothered to bring it down… [Robert 61]

Although this case may have been complicated by operational factors, for the participant is clearly important that ongoing individual care needs were met in these clinical discharge waiting areas:

Consideration needs to be taken into what the effects of discharge will have on peoples’ regime, you know when you are in hospital you get a very strict regime on timings, they wake you up to give you the medication then suddenly they sign you out the hospital and then wash their hands of you, even though you’d not gone out the door… [Robert 61]

### 4.2 Theme 2: Coping at home after discharge from hospital

Many participants were concerned about factors influencing their recovery and the challenges of living with the symptoms of BTI during the early post‐discharge period. This section will explore participants experiences surrounding the following two sub themes: (a) optimising your own recovery at home and (b) living with symptoms after discharge, identifying challenges and ways of overcoming these in the home environment.

#### 4.2.1 Subtheme 2.1: Optimizing recovery

Participants identified the challenge of self‐managing their recovery and their reliance on others to help them with daily life. For many participants, previously “simple tasks” became almost impossible due to their impeded movement, reduced weightlifting tolerance and restricted mobility. For many, even basic functions such as personal hygiene, elimination and getting dressed was time consuming, frustrating and a painful process:

I wasn't able to really dress myself initially, very difficult to go anywhere below [the] waistline. [Calvin 50]

After regaining independence in their daily activities, participants consistently identified a change in behaviour whereby previously “normal” tasks required more “careful judgement” than before the injury happened.

In the early post‐discharge period, sleep was identified as being an important factor in participants’ recovery. For many participants, optimized sleep was essential to not only their physiological recovery, but their attitude towards recovery and coping with their injury at home:

I’m not totally invalid but [I] feel it more sometimes depending how I sleep. [Karen 70]

For many positioning was a process of trial and error, finding ways to maximize comfort and it is apparent that little or no advice on positioning at home was given to patients prior to discharge:

…we had to experiment with different configurations of cushions and my wife bought me this V shape cushion which is the best one… [Robert 61]

It is apparent that there is substantial disparity in the level and amount of post‐discharge follow‐up and many participants were not advised on processes to seek medical support in the early post‐discharge period. For some, this was frustrating and left them feeling abandoned impacting on their recovery and ability to cope at home:

…it frightened me, and I said ‘look, this isn't right you know, it isn't OK to abandon somebody like this… [Bill 65]

#### 4.2.2 Subtheme 2.2: Living with symptoms after discharge

Most participants described reduced mobility at home in the immediate post‐discharge period. Although many participants noted a regular improvement in mobility, there was “frustration” associated with activities they could not complete for themselves. For some older participants, their reduced mobility exacerbated other movement limiting conditions:

I’m not very active [currently]… I do a lot of walking, I do gardening… so I do quite a bit, but I’ve not been doing that for nine weeks now. I suppose it's the fact that I’m not doing that all my joints are seizing up. [Oliver 62]

This reduced mobility appears to have impacted on some participant's confidence and the fear of falling was clear, particularly for participants who were initially injured in low velocity falls from standing height. Having the correct supportive walking aids at home appears to negate the risk and confidence issue:

I had a stick [from the physiotherapist]… when you have a stick you feel more confident. [Richard 78]

One participant described falling at home and injuring his wrist whilst protecting his chest from further injury.

I tripped and fell on the floor and I think I might have fractured my wrist… I don't think it helped when I fell on the floor… I was trying to protect my ribs. [Henry 60]

Severe acute pain in the early post‐discharge phase of recovery was identified by all participants as a substantial challenge to overcome. Many participants reported experiencing severe acute pain which substantially affected their coping ability:

I was in really acute pain and occasionally as I laid in bed it went off but as soon as I tried to move or tried to get up the pain returned and it took some time to get rid of it again like, two or three hours. [Reg 77]

The pain was often exacerbated by voluntary and involuntary functions like laughing and sneezing:

Laughter you can control because you don't want to laugh because you're not very happy anyway so that was easy. But if you have to sneeze… that was very, very difficult. [Calvin 50]

Several participants described changes in the location and character of their pain during the early post‐discharge period. They considered this to be part of the normal progression of pain and were not prepared for the signs of pain with a potential neuropathic component:

…then it started itching across my back and I mean really itching… and burning and I thought, ‘God, I don't like the combination here’… [It is] definitely a different pain, entirely different pain to that which was the fractured ribs and that scared me and it's still there now… [Bill 65]

### 4.3 Theme 3: Using pain relief at home

For all participants, the management of pain with analgesic agents was an important component of recovery in the early post‐discharge period. In this theme, participants identified important aspects of medicines management that impacted on their concordance and recovery.

#### 4.3.1 Subtheme 3.1: Medication supply

Several participants were discharged with insufficient supply or without any medications making the process of continuing care at home very difficult:

…they said it was very important to manage the pain but then they only gave me 3 days’ worth of Tramadol, so I ended up having to go to my GP, I was discharged on the Friday, went to my GP on the Monday to ask for a prescription for Tramadol… I thought it was a mistake … [Robert 61]

#### 4.3.2 Subtheme 3.2: Medication Safety

The safe use of medication was identified by several participants and the potential danger associated with these medications was highlighted by one participant who accidently overdosed on opiates:

…I went back to bed and didn't feel very well and ended up coming back in [to hospital] because I’d overdosed on the painkillers, which was Morphine… they discharged me without the medication because they wanted to get me out of that bed…so I didn't come home with any medication. Luckily, I had some at home when I arrived home the first thing I did was take painkillers and I overdosed on it obviously… [Reg 77]

For this participant, their journey through the discharge process did not adequately prepare them to cope at home. They describe being in acute and severe pain without any discharge medications. This resulted in the patient using opioid medication that were not specifically prescribed by the clinical team responsible for their care:

…I presume if I’d have read the instructions or somebody had told me what could happen if I didn't take them correctly I would have done something differently but all I was concerned about was that I was in agony, every time I wanted to go to the toilet I was in agony getting out of bed… [Reg 77]

Not all participants were aware of the addictive potential of the opioid analgesic agents that they were using. For some, weaning off these drugs was instinctive, but no one was given weaning guidance provided by a healthcare professional.

…If they did, I didn't hear it because possibly they may have but there was very little written, there was no written guidance about medication, how to slow down and how to wean… When I came off [the] drugs I was cold turkey and very confused and very fragile and lots of bad dreams… [Lydia 48]

#### 4.3.3 Subtheme 3.3: Side effects and concordance

Many participants described the common side effects relating to opioid usage. For many, the associated constipation was challenging to manage at home and had the potential to impact on their progress of recovery. Interestingly, one participant highlighted that despite having previously experienced these side effects when taking codeine, alternative options were not explored with them during the discharge process:

…They gave me paracetamol and codeine and I said to them I can’t have codeine because it affects me… if I have one tablet I can’t go to the toilet for a week. But he said, what do you want pain or constipation? So, I said constipation. [Karen 70]

Participants highlighted the central nervous system effects of taking opioid analgesics. One participant found that this side effect was beneficial in optimizing their sleep and this became a primary function of taking the medications:

…it makes it easier in some respects because the pain and discomfort isn’t as bad so you can try and get going, but on the other hand they make you very tired and lethargic, so you don’t want to do too much… I’m sure they help me to sleep more than I would have done had I not been taking them [Oliver 62]

## 5 DISCUSSION

This study explored patients’ perceptions of discharge from hospital and the early post‐discharge recovery after BTI. We identified several factors in the discharge process which impacted on these individuals’ post‐discharge recovery and in so doing this adds emphasis to the need for adequate patient preparation for discharge. In these factors are care quality and patient safety issues that need to be managed in the discharge process for trauma patients with BTI in the future. Pain itself was a re‐occurring concept in all interviews, and therefore, pain was a dominant theme throughout. It is important to recognize that these findings although specific to BTI are relevant to all injury patterns in trauma care (e.g. spinal cord injury, traumatic brain injury, limb trauma etc.) and these results will impact on the discharge pathways for all injury groups. This study has used the experiences of BTI patients to identify the wider needs of trauma patients.

Safety is a key component of patient care and the management of risk underpins clinical practice in all areas of health care. The most striking finding in this study was the potential risk of opioid overdose and the apparent lack of preparation for safe use of opioid analgesics that patients experienced in the discharge process. The early post‐discharge period after surgical admission has previously been identified as a potentially vulnerable time for patients who are opioid naïve, unsupervised and may have escalating analgesic requirements (Baird et al., 2017, 2019; Mudumbai et al., 2019). Despite this risk, the rates of opioid overdose in 30 days for surgical discharge were 0.01% (*N* = 134/1,305,715) suggesting that the risk remains low in recently discharged surgical cases (Ladha et al., 2018). Education is an important factor in preparing patients for discharge from hospital and managing risk outside of the hospital setting (Goldsmith et al., 2018b). For these participants, the reality of self‐management in the early post‐discharge period was different to what they had expected. Providing greater information on safe opioid use and weaning advice could negate the risk of complications associated with unsafe opioid self‐management (Bartels et al., 2016; Feinberg et al., 2018; del Portal et al., 2016; Stewart et al., 2019).

Participants highlighted high levels of psychological burden in the post‐discharge recovery period. For many, this burden manifested itself as poor mental well‐being with symptoms of anxiety and depression. In most cases, this is related directly to the experiences of pain and the limitations of the individual's potential for recovery. The level of psychological sequelae after BTI has previously been measured quantitatively with reports of high levels of poor mental function in both females and younger injured people (Marasco et al., 2015). The psychological burden after BTI has also been touched on in a previous qualitative interview study (Claydon et al., 2017). In this study, there were many similarities in the in the cause and presentation of psychological burden with the findings of this study.

Our participants identified the need for an integrated interprofessional approach to discharge planning and execution as an integral part of optimizing recovery in the early post‐discharge period (Hesselink et al., 2012, 2013, 2014). Where process issues arouse, this commonly involved issues with communication both between professions and with the participants themselves (Goldsmith et al., 2018a, 2018b; Hesselink et al., 2012; Sleney et al., 2014). Interestingly, in our study no participants identified nursing input in the discharge planning process. As the process of nursing care is the only constant throughout these trauma patients’ hospital admission, it may be that they perceive the role of this professional group differently (Krook et al., 2020). Alternatively, even though discharge may be part of the nurse's role, participants did not experience any nursing input for another unidentified reason. Furthermore, several participants highlighted how the limited discharge education and information they were given often resulted in them being unsure how to manage their own recovery after discharge. The issue of patient comprehension of discharge instructions has been highlighted in emergency care. In one study, of 49 patients discharged from a single Emergency Department, 31% (*N* = 15) reported needing further clarification about their diagnosis and aftercare (Zavala & Shaffer, 2011). It is only through effective communication and engaging patients in the decision‐making process that there can be a consistently shared mental model in the discharge process.

Overall, these findings are important for future care planning in the UK NHS as there is a need for greater patient follow‐up and rehabilitation after trauma, but without a commissioning driver, there will always be a disconnect that will leave these services underfunded (Kettlewell et al., 2020). In the meantime, it is important to consider how a patient pathway can be used to optimize the transfer of care responsibility from the secondary care setting to primary care providers (Waring et al., 2014). Although this will require greater integration of electronic patient records, it seems likely that the introduction of a “trauma patient passport” will help transfer important patient information and circumvent challenges in different healthcare systems. As a component of pathway‐based care, patient passports will improve quality and safety in all levels of post‐hospital discharge trauma care. This potential method to improving transition by integrating patient information in an accessible way has been successful in chronic disease management spanning both primary and secondary care (Philip et al., 2019). Further research is needed to understand the feasibility and acceptability of this potential intervention in this population.

### 5.1 Strengths and limitations

A strength of this research is the variation in the sample from a perspective of geographical location and the number of recruiting hospital sites. This allowed us to provide a broad picture of the complexities of recovery after BTI. Another strength is in the qualitative design which allowed a deeper understanding of the challenges experienced by this patient group through the rich data sources in this study.

During the sampling for this qualitative study, it was challenging to get younger participants to take part. The youngest participant was 48 years old, and many participants were aged 60 years or above. This leaves the potential for the views and perceptions of younger people with BTI to be missing in this study. Despite this limitation, the exploration of experience in this sample of participants who were predominately older (>60 years) is valid, as previous research has highlighted the need to explore the experiences of BTI in this older population (Baker & Lee, 2016; Cubitt et al., 2019). Although the findings reported in this study are not generalizable to the BTI population, it is likely that the experiences of these participants are applicable to other BTI patients and the quality and safety issues identified in this study are not unique to this sample alone.

The duration of interviews varied substantially between participants with one interview lasting 12 min in length. Whilst the short duration of this interview may be considered a limitation, this study aimed to conduct focussed interviews last between 15–20 min each. Where participants had more to discuss, then this was encouraged but in the case of this 12‐min interview, the participant had provided all the information relating to their experience that they wanted to provide and did not want to elaborate further.

This study was ongoing when the COVID‐19 pandemic started in the UK. Although it was not possible to investigate how COVID‐19 had influenced participants recruited into this study, it remains important to recognize and acknowledge how the COVID‐19 pandemic impacted on these participants. These potential impacts include those relating to social isolation, mental well‐being and physical illness (Baker & Clark, 2020).

## 6 CONCLUSION

This qualitative study explored patients’ perception of the hospital discharge process and their early post‐discharge recovery after BTI. Whilst the burden of injury remains great, from a patient perspective, there are significant quality and safety risks associated with leaving hospital without adequate preparation. The trauma interprofessional team needs to consider further how a discharge pathway can be developed which aims to manage risk and optimize patient self‐management of recovery in the early post‐discharge period. This is particularly important in pain self‐management which was key to all participants in this study and posed the greatest risk to patient safety.

## CONFLICT OF INTEREST

The authors have no conflicts of interest to disclose.

## AUTHOR CONTRIBUTIONS

EB, AX, CN, PH, GL: Conception and design, or acquisition, analysis and interpretation of data; manuscript drafting or revision; final approval of the version; and accountable for all aspects of the work.

## ETHICAL APPROVAL

Ethical approval was granted by the “Hampshire A” South Central Research Ethics Committee in June 2018 (ref: 18/SC/0230). Although informed written consent was gained during recruitment to the main study, participants included in this qualitative study were re‐consented prior to data collection and further participant information was provided. Participants were given opportunities to ask questions both pre‐ and postinterview, and time was taken to ensure that the participant was comfortable with the process and interview content prior to closing the interview. Where required, participants reporting ongoing physical or psychological problems were sign posted to their local primary care clinician for further assessment. Participants were offered a £10 shopping voucher as a gesture of thanks for the time taken to participate in this study.

## Supporting information

Supplementary Material

## Data Availability

The data sets generated during and/or analysed during the current study are available from the corresponding author on reasonable request.
