# Supplementary material for: The processes of hospital discharge and recovery after blunt thoracic injuries: The patient’s perspective
Source: Nurs Open. 2021 May 18;9(3):1832–43. doi: 10.1002/nop2.929 (PMC8994942; doi:10.1002/nop2.929)
Supplement: Supplementary file 1 — Supplementary Material [file NOP2-9-1832-s001.docx]

| **Main Theme:** | **Sub-theme:** | **Codes:** | **Example quotations:** |
| --- | --- | --- | --- |
| Challenges within the discharge process | Suboptimal care co-ordination within the interprofessional team | Issues in care co-ordination | *“So I had to wait then until they sent somebody to the house and put one of these things on the bed so you can grab hold and pull yourself up. Once that was sorted, I was alright…”* [Richard78] *“…in the end I phoned a chap I know, an ex-client, he runs a chair lift company, a medical supply company so £500 later and I have a chair lift installed…”* [Bill 65] *“…it was a Friday so, they said you’ll need to go to your GP, well my GP requires 5 days, um, the hospital records didn’t update in time on the Friday , didn’t show for some reason, the surgery couldn’t access it, so it was Monday, so yes I went home on Friday midday I suppose and I was taking 2 paracetamol probably every 4 hours to be honest.”* [Bill 65] |
|  |  | Issues in communication | *“…we’ve got… different consultants coming up depending on the injuries, so I had two sets of people coming to see me, the anaesthetist was sorting out pain management and then the surgical people were talking about it as well…”*  [Robert 61] *“…the anaesthetic team said yes you can go. Half an hour later the nurse in charge said no you can’t… ‘they’d got no right to say you could be discharged it’s down to the surgical team’. So, I had to then wait for the surgical team to come and give the OK. So, it was all a bit vague.”*  [John 62] |
|  |  | Understanding of discharge reasoning | *“Well basically they told me there’s not a lot they could do because the broken ribs had got to take time to heal themselves… in all fairness I suppose the hospital looks at it from the point of view they can’t do any more for you other than give you medication which you could take at home.”* [Reg 77] *“…it was purely based on the mobility aspect at no point did anybody tell me what comes next…”* [Bill 65] *“…they said I could go home the day before, but I said I can’t go because there’s nothing to help me in the house…”* [Henry 60] |
|  |  | Lack of patient and family involvement | *“For three or four days my blood pressure was really low and that happens when I lie in bed for a long time my blood pressure just goes super low. I kept telling them, but they were nervous… They didn’t want to let me go.”* [Lydia 48] *“…I said well I don’t really think I’m able to go home because I can’t get out of bed and my wife also can’t help me because she’s not very well herself. They weren’t interested they just wanted to get me out and about and that was it.”* [Reg 77] |
|  | The patient’s expectations of the discharge process | Sharing of discharge information | *“It was at breakfast and they just said how are you feeling today and I said no fine, I think I’m OK to go today …I organised someone to come and pick me up… The nurses came round and then she was like ‘oh right I see you are going; I’ll just go and get your drugs for you and what you need’. [I] signed a couple of forms and I didn’t see anybody else again.”*  [Sally 52] *“They just managed it day by day and said basically because I wanted to get home that they said just see how you get on and we’ll take it day by day.”*  [Oliver 62] |
|  |  | Insufficient written advice and guidance | *“They told me a lot of things which I actually wish they had written, well I had to make them to repeat it many times because I couldn’t digest it and I think if I’d had something written down with a lot of the stuff that they told me it would be helpful…”* [Lydia 48] *“I was getting a visit from a pain manager every day and I can really remember one on the Thursday before I left on the Friday. [They] gave me the most information about what it would be like going home to take whatever painkillers I needed and sleep, which was really good advice, the message about sleeping.”* [John 62] |
|  |  | Being unprepared for early self-management | *“I think when you leave hospital you are still in shock… I didn’t realise what the pain levels [would be like] … I think I was in denial about a lot of things.”* [Lydia 48] *“You do have a bit of apprehension actually at that point because when you are in hospital you are in a safe place if something goes wrong.”* [Calvin 50] |
|  |  | Leaving hospital | *“I was sent from the ward down to a discharge lounge and it took an hour and a half to get off the ward, um after the doctor had seen me… It was made worse by the fact that the discharge lounge people had told me that my medication was ready after 3 hours but nobody from the pharmacy could be bothered to bring it down…”* [Robert 61] *“Consideration needs to be taken into what the effects of discharge will have on peoples’ regime, you know when you are in hospital you get a very strict regime on timings, they wake you up to give you the medication then suddenly they sign you out the hospital and then wash their hands of you, even though you’d not gone out the door…”* [Robert 61] |
| Coping at home after discharge from hospital | Optimising your own recovery at home | Depending on others | *“…there was enough people where I wasn’t on my own. If I’d been on my own that would have been a different story.”* [Calvin 50] *“…if I’d been by myself, I would have been in a really black, black, black lonely desperate space.”* [Lydia 48] *“…my wife is disabled, and she couldn’t help me, she couldn’t even lift me out or help me to get out of bed or do anything at all.”* [Reg 77] *“…Oh I would never have coped at home. My husband has vascular dementia and that’s why my daughter and son in law and granddaughter live with us to help.”* [Karen 70] |
|  |  | Daily life and activities | “*I managed to get out the second week and do my own shopping…I’ve carried on as normal but I’m a bit slower at it. I’ve got to judge things now a bit more carefully…”*  [Stephen 70] *“…I was scared to have a shower that first night in case something happened, and I fell or something because I was a bit shaky on my feet…”* [Stephen 70] *“They gave me a [urine] bottle to take home because I couldn’t get out of bed, I said well I need something and that’s all they did give me was a bottle.”*  [Reg 77] |
|  |  | Optimising sleep and positioning at home | *“I definitely couldn’t lay on my side and sleeping was a nightmare because I had to be flat on my back… When you are flat on your back in the same position you inevitably get uncomfortable and you want to move but you can’t. So, I had a lot of sleepless nights basically.”* [Oliver 62] *“I came home and spent most of the time sitting up in a chair for probably two weeks I have to say.”* [Calvin50] *“…we had to experiment with different configurations of cushions and my wife bought me this V shape cushion which is the best one particularly now everything has eased a little bit…”* [Robert 61] |
|  |  | Impact of extra-thoracic injuries | *“…[I had] 6 fractured ribs, now with a boot on trying to lift my foot, get on the bed, off the bed where the only way you can do so is by using your shoulders was very uncomfortable…”*  [Bill 65] |
|  |  | Accessing follow-up care | *“I had an appointment on the 20th first of all, that wasn’t to do with ribs though that was head injury, and my son took me to that.”* [Temi 71] *“…it frightened me, and I said ‘look, this isn’t right you know, it isn’t OK to abandon somebody like this…”*  [Bill 65] |
|  | Living with symptoms after discharge | Mobility issues | *“I’m not very active [currently], which I normally am. I do a lot of walking, I do gardening… so I do quite a bit, but I’ve not been doing that for nine weeks now. I suppose it’s the fact that I’m not doing that all my joints are seizing up.”* [Oliver 62] *“…it was fear of another fall… as I live alone…”*  [Bill 65] |
|  |  | Shortness of breath | *“…a few days after I got home was that I suddenly started to get quite breathless… I did come close to collapsing. While I’m in the house if I get short of breath, I need to sit down, I can because there are chairs everywhere but I was coming up from the car…”*  [John 62] |
|  |  | Fatigue | *“… [I felt] very tired and lethargic but I think that’s a lot down to the painkillers.”* [John 62] *“…I still find even though I’m off the painkillers now, I do get tired very quickly and taking naps much to my wife’s disgust.”* [Oliver 62] |
|  |  | Pain | *“… [pain is] just an ongoing thing now… they did say this tenderness could take 12 months, each [person] is different.”* [Sally 52] *“I have a high threshold of pain, but this was something I’d never experienced…”* [Karen 70] |
|  |  | Aggravated pain | *“Laughter you can control because you don't want to laugh because you’re not very happy anyway so that was easy. But if you have to sneeze… that was very, very difficult.”* [Calvin 50] *“Then I realised the pain wasn’t only in my back it was right around the side of me to my sternum and I had as much pain in my sternum as I did in my back.”* [Karen 70] |
|  |  | Neuropathic Pain | “…I felt quite numb which I never said to anybody when I was in hospital, I just thought oh it’s a muscle…” [Karen 70] *“…then it started itching across my back and I mean really itching… and burning and I thought, ‘God, I don’t like the combination here’… [It is] definitely a different pain, entirely different pain to that which was the fractured ribs and that scared me and it’s still there now… everybody said I had shingles”* [Bill 65] *“…a certain period afterwards I got all this really bad burning inside and [I] might have also been coming off the meds, I felt the feeling, the sensation of being burnt, like really having an iron or something inside my skin burning inside.”* [Lydia 48] |
| Using Pain relief at home | Medication supply | Taking medication home | *“I don’t think they sent me home with anything. They might have done.”* [Temi 71] *“Well I had to go and see the doctor, obviously the doctor had got the letter, but they wouldn’t prescribe it without seeing me cause it’s a controlled drug…”* [Robert 61] |
|  |  | Accessing further medications | *“…they said it was very important to manage the pain but then they only gave me 3 days’ worth of Tramadol, so I ended up having to go to my GP, I was discharged on the Friday, went to my GP on the Monday to ask for a prescription for Tramadol… I thought it was a mistake and I kept looking in the bags, surely I’ve missed something you know, meant kept looking in the bag, couldn’t see it but it wasn’t there…”* [Robert 61] *“you’ll have to pop down… I can’t drive, I can’t walk, ‘why can’t you walk?’, I said ah… OK, [I] explain…where the 9 fractures were [was] making transport and moving around very difficult …”* [Bill 65] |
|  | Medication safety | Opioid overdose | *“…I went back to bed and didn’t feel very well and ended up coming back in [to hospital] because I’d overdosed on the painkillers, which was Morphine… they discharged me without the medication because they wanted to get me out of that bed…so I didn’t come home with any medication. Luckily, I had some at home when I arrived home the first thing, I did was took painkillers and I overdosed on it obviously…”* [Reg 77] *“…I presume if I’d have read the instructions or somebody had told me what could happen if I didn’t take them correctly I would have done something differently but all I was concerned about was that I was in agony every time I wanted to go to the toilet I was in agony getting out of bed and getting there I couldn’t get any help, I didn’t have any assistance in anyway, I didn’t have any aids in any way.”* [Reg 77] |
|  |  | Weaning from analgesics | *“…I’m trying to wean myself off them because I’m not sure if they are addictive, but they certainly make you very fuzzy…”* [Oliver 62] *“…If they did I didn’t hear it because possibly they may have but there was very little written, there was no written guidance about medication, how to slow down and how to wean or sever and there was no written guidelines…”* [Lydia 48] |
|  | Side effects and concordance | Opioid constipation | *“…They gave me paracetamol and codeine and I said to them ‘I can’t have codeine because it affects me… if I have one tablet I can’t go to the toilet for a week’. But he said, ‘what do you want pain or constipation’? So, I said constipation.”* [Karen70] *“I knew what I couldn’t do and could do, and I thought the painkillers might then mask that and prolong things. Nobody has told me to do that I just thought well because I thought I’d rather do without the constipation, do without pain in my stomach. That was almost becoming a little bit more of a nightmare than me just sitting still and not irritating the ribs.”* [Calvin 50] |
|  |  | CNS side-effects of opioids | *“…the problem with codeine is it just makes you really, really drowsy and you know it’s a very good painkiller.”* [Calvin 50] *“…they made me very tired and a little bit fuzzy headed if you like. I’m sure they help me to sleep more than I would have done had I not been taking them.”* [Oliver 62] |
